# Can only poorer European countries afford large carnivores?

**DOI:** 10.1371/journal.pone.0194711

**Published:** 2018-04-27

**Authors:** Ilpo Kojola, Ville Hallikainen, Timo Helle, Jon E. Swenson

**Affiliations:** 1 Natural Resources Institute Finland (Luke), Rovaniemi, Finland; 2 Rovaniementie 1456, Aska, Finland; 3 Faculty of Environmental Sciences and Natural Resources Management, Norwegian University of Life Sciences, Norway, and Norwegian Institute for Nature Research, Trondheim, Norway; Michigan Technological University, UNITED STATES

## Abstract

**Background:**

One of the classic approaches in environmental economics is the environmental Kuznets curve, which predicts that when a national economy grows from low to medium levels, threats to biodiversity conservation increase, but they decrease when the economy moves from medium to high. We evaluated this approach by examining how population densities of the brown bear (*Ursus arctos*), gray wolf (*Canis lupus)*, and Eurasian lynx (*Lynx lynx*) were related to the national economy in 24 European countries.

**Methodology/Principal findings:**

We used forest proportions, the existence of a compensation system, and country group (former socialist countries, Nordic countries, other countries) as covariates in a linear model with the first- and the second-order polynomial terms of per capita gross domestic product (GDP). Country group was treated as a random factor, but remained insignificant and was ignored. All models concerning brown bear and wolf provided evidence that population densities decreased with increasing GDP, but densities of lynx were virtually independent of GDP. Models for the wolf explained >80% of the variation in densities, without a difference between the models with all independent variables and the model with only GDP. For the bear, the model with GDP alone accounted for 10%, and all three variables 33%, of the variation in densities.

**Conclusions:**

Wolves exhibit a higher capacity for dispersal and reproduction than bear or lynx, but still exists at the lowest densities in wealthy European countries. We are aware that several other factors, not available for our models, influenced large carnivore densities. Based on the pronounced differences among large carnivore species in their countrywide relationships between densities and GDP, and a strikingly high relationship for the gray wolf, we suggest that our results reflected differences in political history and public acceptance of these species among countries. The compensation paid for the damages caused by the carnivores is not a key to higher carnivore densities, but might be necessity for the presence of large carnivores to be accepted in countries with high GDP.

## Introduction

Many animal populations are facing extinction risk caused by human impacts. Traditionally, economic growth has been regarded to threaten biodiversity conservation [1]. A major challenge to biodiversity conservation is to facilitate the protection of species that are valued at a global scale, but have negative value at a local scale, e.g., due to public fears and damage to livestock [2]. Large carnivores often cause economic damage and, conversely, the intensive land use often associated with economic growth is deleterious for large carnivore populations [3–5]. Globally the number of threatened mammal species by country is correlated with high socioenomic factors [6]. On the other hand, biodiversity loss and poverty can be interrelated, whereby conservation might help tackle poverty through, for example, generating jobs in wildlife tourism [7].

One of the classic approaches in environmental economy is the environmental Kuznets curve, derived from Kuznets [8]. The curve predicts that when a national economy grows from low to medium levels, threats to biodiversity conservation increase, but they decrease when the economy moves from medium to high [1, 9–11]. There are several natural and human-mediated factors influencing large carnivore populations and population densities. One human-mediated reason explaining variations in population densities might be public economy, when it is associated with individual humans’ possibilities to minimize livestock depredation and risks to human safety.

In many European countries, both the economy and large carnivore populations are stronger than they were some decades ago [12]. Here we examined how current population densities of the three most widely distributed European large carnivores, the brown bear (*Ursus arctos*), gray wolf (*Canis lupus*), and Eurasian lynx (*Lynx lynx*), are related to national economies in Europe. If the environmental Kuznets curve applies to these species, their population densities would be lowest at the mid-range of gross domestic product (GDP).

## Material and methods

### Data

We obtained mean wolf, bear, and lynx population abundance estimates for 2008–2011 from 24 European countries, based on expert estimates [13]. The availability of suitable habitat, a key factor for successful conservation of large carnivore populations, varies widely among countries. However, only a portion of the land area is suitable habitat. Therefore, we assumed that forest area was representative of the area of suitable habitat and per capita gross domestic product (GDP) was representative of the national economy. In this study, we treated population size [13] per land area of the country (i.e. population density as animals/1 000 km^2^) as a dependent variable. We also used this data compiled by the European Commission [13] to obtain the existence of systems for compensation of depredation caused by large carnivores. We obtained data on land area, the forest area, and GDP in 2011 for each country from World Bank statistics (https://data.worldbank.org/)Due to cultural similarities and differences between countries, some pseudoreplication could be present without taking a random factor of country group into statistical models. We divided the countries into three groups: 1) Nordic countries, 2) eastern European countries, and 3) the remaining European countries (Western Europe, Nordic countries excluded).

### Statistical analysis

By treating population density as a dependent variable, we fitted a linear model with the first- and the second-order polynomial terms of GDP (gross domestic product) for each species separately (brown bear, wolf and lynx). Then we did this with two independent variables, GDP and the proportion forest. The country group was entered as a random factor and the existence of a governmental compensation system as a fixed factor to all models. The country group or the existence of a compensation system did not affect densities significantly (*p* > 0.10) in any model, and therefore, they were excluded from the final models.

The models were built after log-transformation of population density, because the log-transformed distributions fit better, and the transformed values were closer to the normal distribution. We presented parameter estimates and tests in the log-transformed scale, but the predicted values after transforming them back to the original scale. The biases caused by the transformation to the original scale were corrected using an empirical bias correction presented by Snowdon [14]. The correction was based on the ratio of the observed mean of the response and the mean of the exponent of the predicted values.

**Fig 1:**
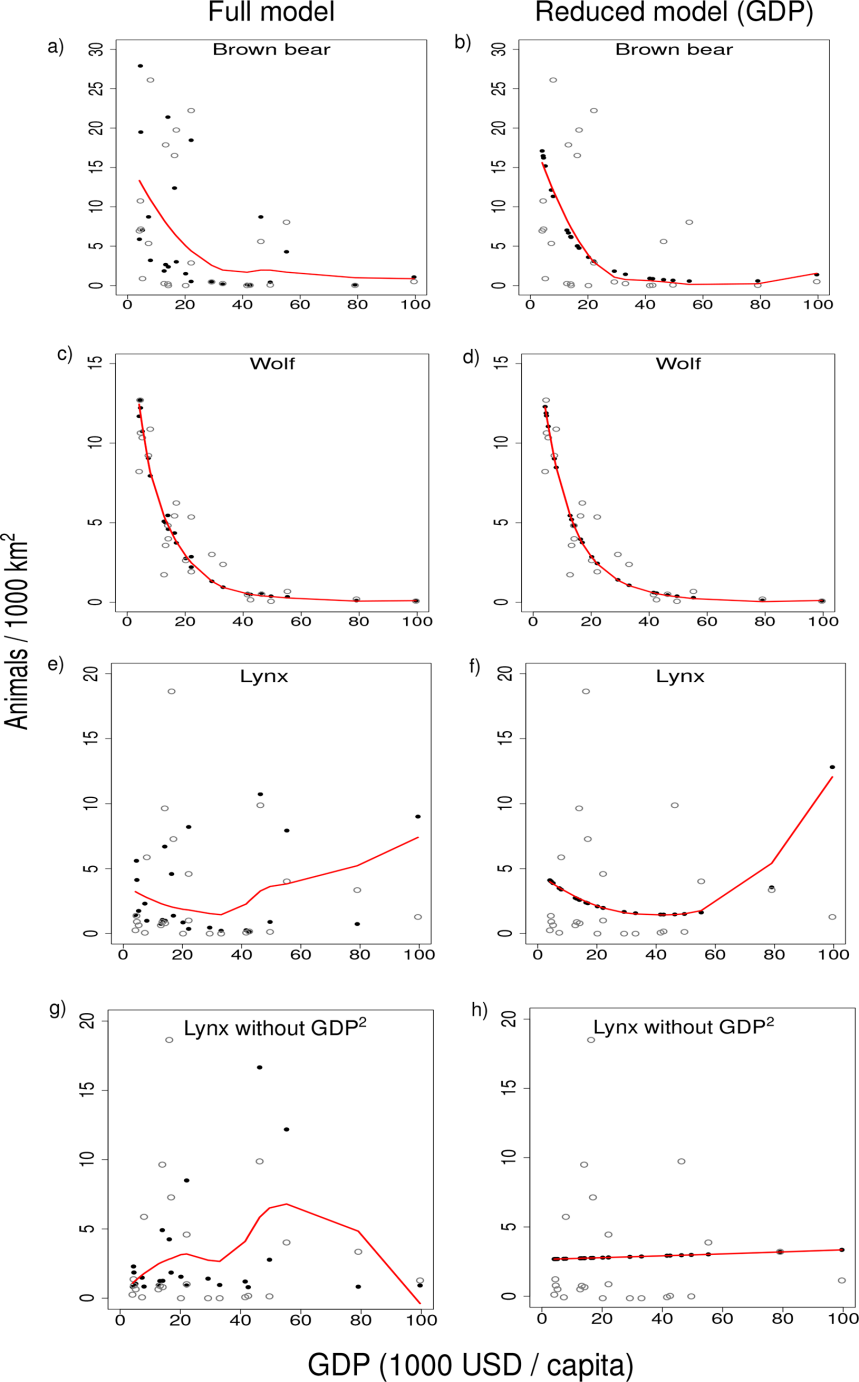
Relationships of per capita gross domestic product (GDP) and population densities of three large carnivore species in Europe. The full model include both forest proportion and GDP as independent variables, the reduced model only GDP. For the lynx, models without the second-order term (GDP^2^) are also shown. Predicted values are shown as black circles and observed ones as open circles. The red smoothed curves are based on the predicted values.

The basic form of the linear models could be described as:

$$Log\left(population\;density\right)=constant+\beta_{1}GDP+\beta_{2}GDP^{2}+\beta_{3}forest\;proportion+\varepsilon ,$$

where *β*_1_, *β*_2_ and *β*_3_ are the fixed coefficients, GDP denotes the gross domestic product per capita, forest proportion denotes the proportion of forest area of the total land area_,_ and *ε* is residual variation. If the second-order term of the GDP influenced the parabola shape of the predicted curve in a well-fitted model with the GDP as the predictor, the Kuznets curve would be supported. For the lynx, we built post hoc models also without GDP^2^ to evaluate the effect of GDP^2^, because the scatterplot with the second-order term (Fig 1F) indicated an increase in population density from medium to high GDP.

The models were computed using R function lm [15]. In premodeling, the random factor of country group (western and eastern Europa, Nordic countries) and the existence of a compensation system were included in the models by using R package lme4, and its function lmer [16]. The variances of the country group were tested using R package lmerTest and its function rand [17].

## Results

Increasing GDP was related to lower densities of brown bear and wolf (Fig 1).

Models both with and without forest proportion supported this observation (Fig 1). However, the variation in lynx density seemed to be virtually independent of GDP (Fig 1).

Based on the adjusted r-squares, the full model accounted for 81.8% and the model without forest proportion 82.3% for wolf densities, 32.9 and 10.4% for bear densities and 20.3 and 4.2% for lynx densities, respectively (Table 1).

**Table 1:** The nonadjusted and adjusted r-squares, parameter estimates, and tests for the two linear models (full models and reduced models without proportion of forest) explaining the population density per 1000 km^2^ of brown bears, gray wolves, and Eurasian lynx in European countries. Two alternative models are presented for the lynx (Lynx 1 and Lynx 2), to define the effect of the second-order term of GDP (with and without GDP^2^).

Variable	Full model			Reduced model		
	Estimate (SE)	p-value	*R*^2^	Estimate (SE)	p-value	*R*^2^
**Brown bear**			0.416/0.329			0.182/0.104
Intercept	-1.46 (1.58)	0.364		2.03 (1.14)	0.090	
GDP	-0.12 (0.07)	0.085		-0.19 (0.07)	0.102	
GDP^2^	1.59e-3 (0.68e-3)	0.030		0.87e-3 (0.73e-3)	0.240	
Forest prop.	11.12 (3.93)	0.010				
**Wolf**			0.842/0.818			0.838/0.823
Intercept	2.62 (0.49)	<0.001		1.99 (0.33)	<0.001	
GDP	-0.11 (0.20)	<0.001		-0.09 (0.20)	<0.001	
GDP^2^	0.56e-3 (0.21e-3)	0.014		0.40e-3 (0.20e-3)	0.075	
Forest prop.	0.82 (1.21)	0.507				
**Lynx 1**			0.307/0.203			0.049/0.042
Intercept	-2.71 (1.42)	0.070		1.30 (0.88)		
GDP	-0.12 (0.06)	0.054		-1.91 (0.07)		
GDP^2^	0.12e-3 (0.61e-3)	0.049		0.11e-3 (0.56e-3)		
Forest prop.	9.61 (3.52)	0.013				
**Lynx 2**			0.155/0.074			0.000/0.000
Intercept	-3.10 (1.51)	0.053		0.70 (0.65)	0.918	
GDP	-0.00 (0.02)	0.892		-0.00 (0.18)	0.891	
Forest prop.	6.91 (3.53)	0.064				

Models without the second-order term (GDP^2^) accounted for only slightly less of the variation in lynx density (ΔAIC = 2.78) [18] than models with GDP^2^, due to a small increase in densities from medium to high GDP (Fig 1, Table 1).

Several factors may contribute large carnivore densities, but it is noteworthy that GDP alone accounted for about 80% of the variation in wolf population densities and did not leave explanatory room for the other independent variable, proportion of forest, in the model (Table 1). For the brown bear, on the other hand, proportion of forest had a greater contribution to explaining densities than GDP (Table 1). The proportion of forest and GDP accounted for 30%, but GDP alone for only 10% of the variation in bear densities (Table 1). Although the proportion of forest was significant in the first model for lynx (Table 1), the adjusted r-squares in the lynx models suggested that the independent variables, GDP and forest proportion, had no clear contribution to the country-specific lynx densities in Europe. For the brown bear model, predictions for the forest proportions of 0.3, 0.5, and 0.7 were population densities of 0.28, 2.58 and 23.92 brown bears 1000 km^-2^, respectively. The predictions for lynx based on the first model (with the second-order term of GDP) were 0.27, 1.87, and 12.77 lynx 1000 km^-2^ with forest proportions of 0.3, 0.5, and 0.7, respectively.

## Discussion

Our analyses demonstrated that large carnivore densities in Europe generally are lowest in the wealthy countries. However, the relationship between GDP and population density clearly varied by carnivore species. The relationship was very strong for the gray wolf, significantly weaker for the brown bear, and did not exist for the Eurasian lynx. These patterns are likely explained by the public image of different carnivore species and political history in Europe.

We did not find any indication of an environmental Kuznets U-shape curve between large carnivore density and national economy in Europe. On the contrary, we found a strong negative correlation between GDP and wolf density, a moderate negative correlation between GDP and bear density, and no correlation between GDP and lynx density. If the recent expansion and increase of wolf populations in Western Europe continues [12] or the rank between national economies changes, this pattern may also change in the coming decades. Brown bear and lynx densities will change more slowly, especially bear densities, owing to the species’ low rates of reproduction and dispersal [19].

Wolves cause the highest per capita losses among carnivores to the sheep and domestic reindeer (*Rangifer tarandus*) industries on the European scale [13,20]. Bears and wolves may attack humans too, but attacks are exceptionally rare [21–24]. The fear of wolves is common due both to real and perceived threats [22,23].

In Europe, wolf population numbers are generally largest in the eastern parts of the continent [12,25]. One potential reason for this is associated with political history. Motivational aspects of wildlife management might be linked to property rights and landownership [26]. For example, the rights to hunt ungulates, the primary prey of wolves, are bound to landownership in Scandinavia, Finland, and Germany [27–30] but not in many former socialist countries, such as the Baltic countries and Poland [31,32]. Mean wolf densities in the Baltics and Poland are considerably higher than in Scandinavia, Finland, and Germany, where ungulate hunters may potentially be motivated to control the wolves as competitors for ungulates. This might be especially true for Scandinavia and Finland, where mean ungulate biomass is low and where producing food is still a major focus of ungulate management [33]. In Scandinavia, acceptance of the illegal hunting of large carnivores is high in areas with strong hunting traditions [34]. Furthermore, citizens of some former socialist countries might have had fewer means to control large carnivore populations than in western democracies, due to limited access to effective firearms.

Another potential reason for the present distribution pattern of large carnivores regards the geography or source populations, especially those within the former Soviet Union, which provided continuous dispersal to other Eastern European countries. Political changes in former socialist countries may also have affected large carnivore populations. In Poland, lynx mortality due to poaching was higher during the low GDP years following the collapse of communism and lower in subsequent years, when GDP had risen to a higher level [35]. In addition, different species may react differently to economic and social perturbations associated with the drastic changes in Eastern Europe. In Russia, for example, numbers of brown bear and lynx decreased whereas wolf numbers increased, following the collapse of the Soviet Union [36,37].

All large carnivores are mobile and especially the gray wolf has a high dispersal potential and colonizes new areas relatively easily. Recently, wolves have naturally reestablished breeding populations in Finland, Sweden, Norway, France, and Germany [12, 37, 38, 39]. They outnumber brown bears in most Central European and Mediterranean countries, but occur at lower numbers than bears in Estonia, Scandinavia, and Finland (12). Suitable habitat in some Western European countries, such as the United Kingdom, Netherlands, Denmark, and Belgium, is too small and isolated for the establishment of a wolf population and the species was exterminated there several hundred years ago [25]. However, wolves were also exterminated from Scandinavia [38], where human densities are among the lowest in Europe.

Compensation paid for the damages are lowest in poorer countries, even when corrected for per capita GDP (Kojola et al., unpublished data). Compensation for the damages does not unequivocally enhance the conservation of large carnivore populations [40,41], but may be a requirement for large carnivore conservation in wealthier countries in Europe, into which large carnivore populations have recently expanded [12] and where people are therefore less used to their presence. Paying for their presence may promote conservation of large carnivore populations [42, 43], although there is also evidence that predator poaching is influenced more strongly by social than economic factors [44].

Our results reflected differences in political history and public acceptance of three large carnivore species in Europe. Although large carnivores, including the gray wolf, have been increasing recently in many wealthy countries [12], a strikingly high negative relationship still prevails between GDP and wolf densities. Relationships to GDP may weaken in the future if conservation policies will favor higher large carnivore densities in wealthy countries. The ongoing population growth of large carnivores in countries with high GDP will provide a test of how well wealthy countries will manage the conservation of large carnivores. The compensation paid for the damages caused by the carnivores is not a key to allowing higher carnivore densities, but might be necessity for the presence of large carnivores to be accepted in countries with a high national economy.

## Supporting information

S1 Table Data used in this study. (XLSX)
